# Understanding innovators' experiences of barriers and facilitators in implementation and diffusion of healthcare service innovations: a qualitative study

**DOI:** 10.1186/1472-6963-11-342

**Published:** 2011-12-16

**Authors:** Julie Barnett, Konstantina Vasileiou, Fayika Djemil, Laurence Brooks, Terry Young

**Affiliations:** 1 Department of Information Systems and Computing, Brunel University London, Uxbridge, Middlesex, UB8 3PH, UK; 2 Systems Engineering and Human Factors Department, Cranfield University, Cranfield, Bedfordshire, MK43 0AL, UK

## Abstract

**Background:**

Healthcare service innovations are considered to play a pivotal role in improving organisational efficiency and responding effectively to healthcare needs. Nevertheless, healthcare organisations encounter major difficulties in sustaining and diffusing innovations, especially those which concern the organisation and delivery of healthcare services. The purpose of the present study was to explore how healthcare innovators of process-based initiatives perceived and made sense of factors that either facilitated or obstructed the innovation implementation and diffusion.

**Methods:**

A qualitative study was designed. Fifteen primary and secondary healthcare organisations in the UK, which had received health service awards for successfully generating and implementing service innovations, were studied. In-depth, semi structured interviews were conducted with the organisational representatives who conceived and led the development process. The data were recorded, transcribed and thematically analysed.

**Results:**

Four main themes were identified in the analysis of the data: the role of evidence, the function of inter-organisational partnerships, the influence of human-based resources, and the impact of contextual factors. "Hard" evidence operated as a proof of effectiveness, a means of dissemination and a pre-requisite for the initiation of innovation. Inter-organisational partnerships and people-based resources, such as champions, were considered an integral part of the process of developing, establishing and diffusing the innovations. Finally, contextual influences, both intra-organisational and extra-organisational were seen as critical in either impeding or facilitating innovators' efforts.

**Conclusions:**

A range of factors of different combinations and co-occurrence were pointed out by the innovators as they were reflecting on their experiences of implementing, stabilising and diffusing novel service initiatives. Even though the innovations studied were of various contents and originated from diverse organisational contexts, innovators' accounts converged to the significant role of the evidential base of success, the inter-personal and inter-organisational networks, and the inner and outer context. The innovators, operating themselves as important champions and being often willing to lead constructive efforts of implementation to different contexts, can contribute to the promulgation and spread of the novelties significantly.

## Background

The ability to innovate is considered as a major competitive advantage in organisations, enhancing their effectiveness, efficiency, and thus their potential for long term sustainability [1]. The concept has been strongly identified with manufacturing, where innovations concern products and artefacts, while the service sector has, by contrast, been seen as a "laggard" [2]. However, the rapid expansion of the service sector in modern economies and the increasing "servicisation" of many, previously pure, manufacturing industries [3] have shifted the focus of attention to new forms of behaviour and activities, expressed as service innovations.

The healthcare domain, and its ability to implement and diffuse innovations has generated intense scientific interest (for reviews see [4,5]). The need for innovation in service delivery and organisational functions has been emphasised since the early 1980s [6]. Nevertheless, healthcare systems in developed countries continue to encounter considerable difficulties in implementation, and experience major delays in diffusing novel initiatives, despite the perception that healthcare organisations are arguably among the most knowledge-rich and scientifically-based institutions [7].

The imperative to innovate in healthcare has recently intensified under the economic challenges and the increasing demands of an ageing population. In the UK, this has resulted in calls for reform of the National Health Service (NHS), recently crystallised in the *Equity and Excellence: Liberating the NHS *White Paper [8]. This aligned healthcare policy around the Quality, Innovation, Productivity, and Prevention (QIPP) programme initiative with the redesign of health services aimed at improving quality while making significant efficiency savings. The notion of innovation occupies a core position within this reformed policy framework now placed at the heart of the healthcare agenda.

### Conceptualising healthcare innovations

Innovation can be defined as "the intentional introduction and application within a role, group or organisation of ideas, processes, products or procedures, new to the relevant unit of adoption, designed to significantly benefit the individual, the group, the organisation or the wider society" [9] (p.209). Anderson et al. [10] suggest that this definition presents several advantages: firstly, it demarcates innovation from creativity, secondly, it conceptualises innovation as a deliberate effort, aiming at benefit, and thirdly, it highlights the relativity of novelty. Following this definition, Länsisalmi et al. [5] suggested that healthcare innovations typically comprise "new services, new ways of working and/or new technologies" (p. 67). These novelties "are directed at improving health outcomes, administrative efficiency, cost effectiveness, or users' experience and are implemented by planned and coordinated actions [4] (p.582). Healthcare innovations constitute particularly complex outputs, since they frequently combine both product and process novelties, or embodied and disembodied components [11] with diversified levels of materiality or tangibility.

### Theoretical perspectives in healthcare innovation research

A large body of research on healthcare innovation has been strongly influenced and shaped by Rogers' seminal work on diffusion of innovation [12]. Within this approach, healthcare innovations are adopted and diffused more easily when certain conditions are favourable. For example, the perceived relative advantage, the potential to trial and observe the effects of the innovation, and its compatibility with the values, norms and beliefs of the adopting system are all believed to facilitate the diffusion [13]. Furthermore, innovations are more likely to spread when they are consistently supported by key opinion leaders and when homogenous groups of people sharing common values are involved. Social networks and inter-organisational partnerships are also recognized as highly significant forces [13-15].

Other theoretical approaches propose a more complex and often turbulent process of innovation diffusion that follows "a nonlinear cycle of divergent and convergent activities that may repeat over time and at different organizational levels"[16] (p.16). For example, researchers have studied the impact of scientific information on the diffusion of healthcare innovations [17-19] and have found that robust scientific evidence does not necessarily lead to innovation adoption in a linear or direct way. The relative advantage of healthcare innovations, and even the evidence of benefit, is not a judgement rooted in pure rationalistic reasoning, but rather is subject to debate and negotiation. This is partly linked to multi-professionalism [20] characterising healthcare where the different professional groups adhere to and value different types of evidence, and also to the demands stemming from the context in which evidence is embedded and interpreted [21].

Finally, adopting a more systemic approach the theory of disruptive innovation [22,23] has also been proposed as a conceptual framework to enhance our understanding concerning the difficulty the healthcare systems have to lead and sustain innovation. According to this theory, disruptive technological advancements in healthcare are not embedded in corresponding business-model innovations which would allow healthcare organisations to take full advantage of these technological enablers and to deliver 'pure' value propositions to their users. This happens because healthcare organisations, structured historically in the form of hospitals and physicians' practices, conflate fundamentally different business models. For disruptive innovations to become embedded in healthcare, several factors need to be aligned including suitable business models and regulatory reform while the inherent technological characteristics of the change and specifically, its ability to simplify are also critical [22,23].

### The aims of the present study

This study sought to shed further light on the question of process-level innovation and to understand more about why diffusion is apparently so poor in healthcare. By interviewing award winners of service innovations, we sought to understand innovation from inside the organisation by exploring the perspectives of those who drive it. Technology was not excluded from these case studies but accommodating a new technology was not the principle driver for innovation. Specifically, we were interested in examining how healthcare innovators perceived and made sense of their experiences of factors that facilitated or constrained their success. We adopted a critical realist perspective [24] which allowed us to consider how these factors were constructed and interpreted in innovators' accounts. A qualitative study, with semi-structured interviews, was conducted among NHS employees highly engaged with particular innovations.

## Methods

### Sample and recruitment strategy

Healthcare organisations in the UK, which had generated and implemented service innovations and whose efforts had been recognized through an award, were the study population of this research. Specifically, primary and secondary healthcare organisations who were winners of Health Service Journal (HSJ) [25] awards from 2007 to 2009 were invited to participate. The HSJ is the premier weekly journal read by NHS managers and healthcare professionals with a print circulation of 17.680 copies [26]. The organisations submitted an application for inclusion in the contest, and following a favourable assessment, presented their cases to a panel judges in a face-to-face presentation, following which final decisions were made. In this way, we secured a measure of innovation that was external-to-the-innovators and which provided a relatively comparable metric of their success. HSJ awards reasonably affirmed a successful implementation status, at least within a single organisation. Additionally, winning these awards indicated that these innovations were valued positively within the NHS system, and that broader adoption would be judged as desirable. Moreover, a variety of service innovations with different aims and from diverse contexts were targeted; all initiatives were process-based, even though product-based innovation was sometimes part of the overall initiative.

Fifty-one organisations (20 primary care organisations, 30 secondary care organisations, and 1 educational institution in collaboration with the NHS) of HSJ service innovation awards were approached and informed about the study. Twenty four of these expressed an interest in knowing more, and of these 15 agreed to take part in interviews (response rate: 29.41%). Table 1 summarises the participating innovations, providing a short description and the award title for each of them. The classification of the innovations presented in table 1 was developed by the researchers based on the key theme of the innovations and their relationship to the main function of the NHS system, namely the provision of healthcare. Among the participating organisations, 5 provided primary care and 10 secondary care. Thirteen out of 23 HSJ award categories are represented in our sample (see table 1), while the following are not: 1. Acute Healthcare Organisation of the Year, 2. Chronic Disease Management, 3. Communications, 4. Cost Effective Partnership, 5. Implementing NICE Guidance, 6. Information-based Decision Making, 7. Patient Safety, 8. Primary Care Innovation, 9. Reducing Health Inequalities, and 10. Data-driven Service Improvement.

**Table 1:** Classification, award titles, and descriptions of innovations

Innovations directly related to healthcare provision		
***Categories***	***Award titles***	***Short description ***

I. Innovations of specified healthcare provision	1. World Class Commissioning	1. Initiative that developed a timely and coordinated hyper-acute service provision to stroke patients with early care and fast access to services, and educated organisations to implement stroke care according to national guidelines
	2. Clinical Service Redesign	2. Initiative that redesigned the acute stroke service offering rapid assessment and care provision, and rehabilitation closer to home
	3. Mental Health Innovation	3. Initiative that provided evidence-based interventions to chronic respiratory patients helping them to manage their synchronic mental health conditions (e.g. depression)
	4. Best Social Marketing Project	4. Initiative that targeted pregnant women to access smoking cessation services, designed on the basis of their needs

II. Innovations related to the overall organisational function	1. Primary Care Organisation of the Year	1. Initiative that concerned a variety of organisational functions, from several health care provision programmes and sound financial management, to the development of local partnerships and the positioning of the organisation as a leader

III. Innovations related to patients' safety*	1. Acute & Primary Care Innovation	1. A reliability checklist that ensured the conduction and revision of appropriate medical checks in round-wards
	2. Improving Care with Technology	2. A technologically-based innovation that secured adherence to evidence-based guidelines during the blood transfusion process, reducing errors/omissions, paperwork, process time per patient and staff capacity requirements

IV. Innovations of patients' access and reception of healthcare	1. Improving Patient Access	1. Initiative that improved prisoners' access to healthcare services through their involvement and the development of the scheme of "prisoner healthcare representatives"
	2. Patient Centred Care	2. Initiative that applied a patient-centred model of care giving patients choice, involvement and control over their care experience

V. Innovations of educational services	1. Mental Health Innovation	1. A training programme that educated professionals in evidence-based family interventions, increasing their awareness to the needs of carers of mentally ill patients
	2. Skills Development	2. A training programme that educated diabetes diagnosed patients to better self-manage their condition and provided continued professional development to trainers and educators.

**Innovations less related to healthcare provision**		

***Categories***	***Award titles***	**Short description**

VI. Innovations related to human resources	1. Recruitment & Retention	1. A human-resources and workforce development initiative that reformed the recruitment and retention practices through the provision of training programmes and employment opportunities to local unemployed and excluded groups
	2. Workforce Development	2. A workforce transformation programme that prepared staff to provide care closer to home

VII. Innovations related to other organisational functions (e.g. logistics)	1. Good Corporate Citizenship	1. Initiative that introduced a transportation scheme, IT developments and new methods of food procurement and waste management which increased organisational sustainability and supported the local economy
	2. Best Social Marketing Project	2. Initiative that provided free access to leisure facilities to disadvantaged groups of citizens in collaboration with the city council

* Technological products were incorporated

Finally, the people interviewed were directly engaged with the service innovation; in most of the cases, they had been involved with generating the initial concept and had led the processes of implementation and diffusion. All interviews were conducted with one innovator, except for one case (Good Corporate Citizenship; see table 1) where four interviewees took part since they all had contributed to different aspects of the innovation. As the focus of our research was on examining a range of service innovations rather than seeking different perspectives around the same innovation, we chose only to interview a single key informant around each innovation and as part of the interview explored the role attributed to others in directly or indirectly contributing to its implementation and diffusion.

### Data collection and ethical considerations

Semi-structured telephone interviews, lasting around 45 minutes, were conducted at scheduled times during September-October 2010 with key representatives of the healthcare service innovations. Initially, people were contacted by e-mail, informed about the aims of the study and invited to take part in a telephone interview. Prospective interviewees were provided with an information sheet explaining the aims, procedure and ethical aspects of the project, as well as the consent form in which they confirmed their willingness to participate. Latterly, interviewees were able to indicate their agreement with the information disclosed and the validity of our analysis (i.e. testimonial validity [27]) in a pre-publication draft paper.

Interviews included questions about the generation and development of the service innovation, the barriers and facilitating factors experienced, and the wider uptake of the innovation. Rather than directing the interviewees to discuss specific themes previously identified in the literature, we deliberately created open-ended questions. This allowed the interviewees to pick up spontaneously on those factors that were perceived as most important for their innovation and then they were prompted to discuss the issues in more depth. More specifically, the interview schedule was comprised of the following questions:

A. Conception and development of the innovation

Could you please tell me about (name of the innovation)?

Where did the idea come from?

Can you tell me about the process of developing this?

How much opposition/interest did you meet along the way?

How was the service innovation viewed by your organisation/in your local context? Did that change at all over time?

B. Diffusion of innovation

Has your innovation been picked up by others?

How did this come about?

What have they done with it?

What do you think were the main reasons for their interest?

C. Perceived barriers and facilitators of innovation diffusion

What factors do you think have encouraged/constrained its uptake by others?

What do you think are the main reasons for this?

How could this have been different?

Participants also provided information about their role in the organisation and the innovation process as well as their participation in the actual HSJ competition. The study received ethical approval from the Research Ethics Committee of Brunel University London.

### Analytic procedure

A thematic analysis, informed by a critical realist stance [24] was conducted on the transcribed interviews. A critical realist epistemological position offers the possibility of accounting for the ways in which the social world is constructed through language, whilst simultaneously recognizing the existence of an external-to-discourse reality. This reality is constituted by the material and institutional world and is considered to influence the meanings and the discourses which people invoke to construct the objects at stake [28]. The employment of a critical realist perspective allowed us to locate innovators' experiences within the broader material and institutional contexts they operated.

Thematic analysis was considered to be an appropriate technique identifying as it does, "repeated patterns of meaning" across a set of data [29,30] (p.86). An inductive approach with a full presentation of themes was selected and the data were analysed semantically, emphasising the explicit meaning of the themes. In addition to description, an interpretation of the themes is provided.

There were four stages in the analysis. Initially, there was a familiarisation process with the data; then, an initial coding took place, where particular extracts were named and defined. Next, themes and subthemes were developed by aggregating the respective coded segments. The construction of the comparative analytical categories was assisted by the use of computer software [31]. Finally, the themes and subthemes were revised and refined, ensuring that the criterion of internal homogeneity and external heterogeneity [32] was met satisfactorily, and that the themes reflected the data. Initial coding of the data was applied by KV, and the themes and sub-themes were developed, revised and refined by JB and KV.

## Results

Four themes were identified in the analysis and are presented in the following sections: (a) the role of evidence, (b) the role of partnerships, (c) the influence of champions and other human-based resources, and (d) the impact of contextual factors both organisational and external. All of these played a role, alone or in combination, to facilitate or block implementation or diffusion of the innovation.

(In order to protect the participants' anonymity, as they are drawn from a narrow range of award years, after each quote below, the innovation is identified by the extent to which it is related to the healthcare provision-see Table 1 for the relevant categorisation).

### A. The role of evidence

Evidence was considered to play a crucial role. This was visible at multiple time points in the initiation, implementation and diffusion of service innovations. Specifically, there was a focus on quantitative evidence and this was seen to operate in three main ways: (a) as a proof of effectiveness, (b) as a means of diffusion and (c) as a precondition for the initiation of the innovation.

#### A1. Evidence as a proof of effectiveness

"Hard" evidence, in the form of quantitative data, was perceived as the 'gold standard' demonstration of effectiveness, constituting an optimal and credible base for assessing an innovation. It equipped innovators to attest to the usefulness and success of their initiatives, and to persuade prospective adopters that they were valuable. A participant characteristically commented:

"So our outcomes are important to us, because they give us sort of a language by which we can sort of articulate the success of it." (Innovation less related to the healthcare provision)

Even when the innovators considered that "soft" aspects of the impact were valuable and significant for the demonstration of usefulness, they also attempted to corroborate this with numerical evidence. The latter represented the indisputable metric needed to buttress accounts of "soft outcomes".

"All the guys that work for me work for nothing, and the job satisfaction of seeing these guys change and...build confidence...is just absolutely amazing, and I think, if nothing else, if people want to call it soft outcomes-I mean, we have got figures of how we've improved stuff, you know...I can give you some statistics on that, on how we've improved stuff in 2009." (Innovation directly related to the healthcare provision)

Lack of quantitative evidence was seen as a notable shortcoming not only for the sustainability and diffusion of the innovation but also for its initiation. This perception was particularly pronounced where there were other barriers and when the initiative was peripherally linked to the core business of the NHS. In such cases, quantitative evidence that would link the innovation to health-related outcomes was imperative.

"One of the things which we need to expand is the health impact of schemes like this (social inclusion schemes), whilst we have a lot of anecdotal evidence we need to gather empirical evidence to demonstrate the long term benefits. In an increasingly challenging fiscal climate, supporting evidence may encourage organisations to make the investment in this type of development." (Innovation less related to the healthcare provision)

Within certain professional groups, almost notably clinicians, "hard" evidence which would be obtained from scientific methods was construed as the necessary prerequisite for the demonstration of the innovation impact, without which any persuasive effort was doomed to failure. Anecdotal or experiential testimonies were unable to exert any significant influence; scientific data were seen as the only basis for a process of persuasion, and on which prospective adopters could make informed decisions.

"For some reason, with doctors, if you haven't got some data, and maybe a p-value, then it's really hard to convince them that something works. What we've found it is, if we just give it to people and tell them it's a good idea, they don't believe us; whereas, if we give it to people and we show them our chart, where we can show that, when we use the checklist, we have a massive change, and suddenly we've got a p-value, that seems to win hearts and minds." (Innovation directly related to the healthcare provision)

#### A2. Evidence as a means of diffusion

"Hard" evidence was also a sound means of dissemination, both intra-organisationally and inter-organisationally. Encouraging health outcomes based on empirical data led organisations, in some instances, to propose the adoption of innovation to other departments. Moreover, the availability of evidence enabled innovators to neutralise resistance and opposition.

"...So instead of taking an aggressive approach to that, we backed it up with data...So we simply just sent the audit data round, which picked up a few people." (Innovation directly related to the healthcare provision)

Similarly, evidence was seen to help innovators obtain human and financial resources needed to expand geographically and inter-organisationally.

"...and then we actually audited the results of their intervention...and actually evidenced that the team were having an effect on patients' wellbeing, and that led to the team being enhanced staff-wise, and the service then rolling out city-wide." (Innovation directly related to the healthcare provision)

#### A3. Evidence as a precondition

Quantitative data were often seen as a base and precondition for the initiation of the innovation, indicating the need for change or the scope for benefit. Pre-existing evidence, which was available before the initiation of the innovation, was used to argue, justify and bolster the case for the developing initiative and it clarified innovators' incentives and intentions, particularly when resistance was anticipated. In this way, uncertainty was reduced and the stakeholders involved could estimate the potential risks and benefits.

"I personally was surprised that there wasn't more opposition really to closure than that, but I think people were genuinely, staff included, genuinely convinced by the evidence supporting why this was the right move and what it would mean." (Innovation directly related to the healthcare provision)

### B. The role of partnerships

Inter-organisational connections, either formalised as partnerships or loosely linked, constituted an integral part in the process of developing, establishing and diffusing the innovations. In some instances, the partnerships were seen to be part of the essence of the innovation itself.

Existing working relationships between partners' organisations were often identified as the starting point of the innovation and the driving force for its development. Trust and mutual support were vital prerequisites for cooperation, since they ensured that the decisions and commitments made would be adhered to by all parties. The importance of trust was amplified when there was high uncertainty around what would follow.

"But I think why it worked for us was that we had a combination of a good working relationship in an environment where the decisions could be made by those people, so you already had some trust, you already had confidence that partners were going to pull their weight...I mean what the PCT [Primary Care Trust] didn't know was whether people would come and use their offer, but what they did know was that if they gave the city council in our area the money, we'd do with it what we said we'd do with it." (Innovation less related to the healthcare provision)

The building of partnerships was a goal of innovators in their attempt to initiate and establish their services, and the lack of them was often assumed to be a reason for failure of previous initiatives, even when these might be well supported financially. The quote below illustrates this point.

"Then, as we were looking around, we realised we hadn't really got any formal links with other services...and actually, whilst we'd got a good level of resource going into the [name of previous service], we weren't getting the impact that we wanted. So, that's where it started from." (Innovation directly related to the healthcare provision)

Having built supportive partnerships, the innovation gained more chances of sustainability in the long term, since partners represented a securing mechanism, warranting and endorsing the continuation.

Dissemination of service innovations was also seen to be contingent upon partnerships. Partners who had an interest in the novelty were perceived as significant in promoting and publicising the message beyond organisational boundaries.

"...and we've got some very good relationships with voluntary organisations anyway, but to just obviously build on that, do more promotions out in the community, working with organisations in obviously promoting what we're doing and how that could fit into the outside world." (Innovation directly related to the healthcare provision)

Importantly, partnerships were sometimes seen as one of the beneficial side-effects that enabled people to construct a common communication framework and a mutually shared agenda, potentially useful for future interactions and collaborations. Interestingly, in some instances the emergent partnerships themselves were identified as a novel characteristic of the service, usually when the collaborations were believed to be exceptional and rare in the field of healthcare.

"It is the process and, you know, great credit to the PCTs across [city name] to agree to work together. That isn't the same in lots of other areas. And so the structures that were put in place and the way that they worked closely with the clinicians and the wider stakeholder group certainly was innovative." (Innovation directly related to the healthcare provision)

Finally, and in retrospect, success was perceived to have been unlikely without the *a priori *consensus of the involved partners. Proactive engagement and dialogue were vital because organisations had the opportunity to agree on the principal elements of the initiative, and in turn the perceived risks were minimised, the undertaking was legitimised and the partners were committed to support the initiative and to follow the rules.

"...I think, you know, because we had great stakeholder engagement, and we'd started off with a big consensus event where we'd agreed some basic principles that we would abide by, then I think everybody felt comfortable." (Innovation directly related to the healthcare provision)

Proactive engagement with partners was also seen as an effective strategy against future resistance and able to mitigate potential obstructions, particularly when the innovation was perceived to be radical and to diverge significantly from the existing norms.

"So we knew that we'd have to come up with something completely different, so we engaged key stakeholders at the outset... because we involved them at the beginning we didn't receive any opposition." (Innovation directly related to the healthcare provision)

### C. People-based resources

People within and outside the organisations were perceived as particularly significant, either in facilitating or inhibiting the innovation journey.

Most interviewees highlighted the importance of champions, who could be employees in various organisational positions, people in the local community or the users of the innovation. Importantly, the innovators themselves, in being passionate about and committed to their initiatives, ultimately became champions.

The role of top and senior management was critical, since the financial support of the initiative, and thus its sustainability and success, was often contingent upon their decisions. Public espousal of the core ideas of an innovation was also represented as a key resource, able to transfer new knowledge necessary for the advancement of the initiative. The interviewee below, commenting on the significance of champions, said:

"It is important. We're fortunate, we're led from the top, our Chief Executive and our Chairman are very passionate, as are our Trust Board, about sustainability. We've also got an environmental awareness campaign that we started again in the New Year that's just gone. We've now got 147, I think it is, environmental champions across the Trust, that are driving it forward, and we're recruiting all the time. These are obviously voluntary posts, but the passion and the commitment out there is absolutely fantastic. So I don't think it would stop. I think it would take it to new levels, because people are bringing in things that we've never even thought of." (Innovation less related to the healthcare provision)

The users of the novel services could also constitute powerful champions, as they were able to circulate their experience to the local community, thus promulgating the new service and counteracting possible resistances.

"The students themselves who have come through the Academy have demonstrated behaviours which are exemplary and they have been the greatest advocates for the Academy,-the reputation of the Academy has allowed it to grow, we do still have people who are anti the Academy, which is reflective of working with excluded groups, but we have far more supporters." (Innovation less related to the healthcare provision)

Employees were seen as a vital channel for intra-organisational and inter-organisational diffusion, since they can persuade their colleagues informally or influence decisions directly, especially when they occupy key positions. They were considered as powerful advocates when they experienced beneficial results in their daily working routines and regarded the innovation as advantageous. By contrast, employees affected negatively can put up barriers that require effort to surmount through proactive engagement and timely information provision.

"...the way that we did that was to just start off with very small pilots in non-acute areas, and develop a process which was so much better for the nursing staff that they were, you know, they were so delighted to have it that they would, you know, they would work with us and become advocates for us when we went to new clinical areas." (Innovation directly related to the healthcare provision)

Barriers to the implementation and diffusion of innovations were perceived to arise when the innovators and the decision-makers belonged to different professional groups. Different educational backgrounds, organisational roles, and diverse worldviews resulted in different priorities, which could delay or obstruct the spread of innovation. In such cases, innovators had to devote much effort to persuading decision makers of the usefulness of the initiative.

"...sometimes the people who are in charge of the budgets are not necessarily very familiar with clinical priorities. So they might be somebody who's actually an accountant, who's responsible for helping the PCT decide which disease areas and which services to put their money in. So you have to...be prepared to almost educate people about why what you're doing is important." (Innovation directly related to the healthcare provision)

### D. Contextual factors

The context, both intra-organisational and extra-organisational, was also perceived to decisively influence the life-cycle of the healthcare innovations.

#### D1. Intra-organisational context

three basic subthemes were identified, relating to: (a) organisational receptiveness, (b) available resources, and (c) organisational capability to promote the innovation.

##### a. Organisational receptiveness

A series of long-term changes often preceded implementation of the service innovation, especially when the latter was large-scale and system-wide. These changes were believed to prepare the organisation structurally and functionally to receive the novelty smoothly, especially when the impending initiative was complex and multifaceted. In this case, the process of implementation and spread was constructed as an incremental change, embedded in an already changing system.

"...we'd been developing our pathway for stroke and aspects of it, year on year, since 2000, and I think it was that foundation that truly enabled us to respond in the way that we did and deliver that [innovation]." (Innovation directly related to the healthcare provision)

Organisational culture was also perceived to be a critical factor. Specifically, the openness of the organisation to trial new ideas and carry the associated risks was seen as significant, particularly when the change was not triggered by external factors, such as policy initiatives, or an obvious and urgent organisational need.

"...there was an environment of being prepared to take a risk, with the right kind of conditions to support that." (Innovation less related to the healthcare provision)

Equally important for diffusion was the fit between the innovation and the organisational ethos. When innovators' values were perceived to be congruent with prevailing organisational norms and beliefs, the diffusion was facilitated, since the novelty affirmed the cultural organisational orientation. By contrast, when the innovation collided with basic organisational principles, resistance emerged and dissemination was impeded.

"You know, it's just changing-it's a big culture change, and it does meet with controversy and it does meet with people who still feel that prisoners shouldn't have any rights at all, and so you are constantly coming up against that." (Innovation directly related to the healthcare provision)

##### b. Available resources

Sufficient human and financial resources were of paramount importance not only for the proper implementation of service innovations but also for their diffusion to other organisations, sectors and fields of practice. Shortage of resources, or fear of this, could block innovators' efforts and led to stagnation. An interviewee, employing the metaphor of "paralysis", commented:

"It's very clear what we need to do in stroke, and sometimes, just the paralysis is just that the money isn't there to develop early supported discharge." (Innovation directly related to the healthcare provision)

##### c. Organisational capability to promote the innovation

Innovators' own capability to promote their initiatives within and beyond their organisation was considered to facilitate diffusion. Specifically, awards, media attention and the possibility of academic publications were viewed as a powerful means of communicating the innovation to the wider publics.

"When we got that far, and, crucially publicised what we'd been doing-enter awards, produced journal publications-which was very helpful to us. You know, having the picture of the process on the front page of the premier international transfusion journal, things like this.., trying to develop some momentum behind it." (Innovation directly related to the healthcare provision)

Winning awards was experienced as a crucial social recognition in three main ways: firstly, awards were perceived as an external and unbiased validation of the innovation, against which it was difficult for doubters to argue. Secondly, awards were seen to raise the profile of organisation and its reputation of being innovative, which in turn helped it to build further networks and inter-organisational collaborations. It also identified the organisation as an early adopter. Thirdly, innovators regarded the awards as an effective means of promoting an agenda, which in turn could attract further resources.

"...if you do have a good profile nationally, regionally, things come to you. You know, you are invited to participate in things, to be early adopters and, that's for the benefit of the people that we serve really, so it's not just about the glory-it's about being at the table..." (Innovation directly related to the healthcare provision)

Active promotion of the innovation with other organisations was presented as essential and innovators believed that they should communicate and publicise on an on-going basis. In this way an extrovert organisational culture is cultivated. This sharing-behaviour enabled innovators to identify pitfalls and advise prospective adopters of the most promising ways of implementation.

"You know, that's one of the big ethos behind kind of the networks really is, it's around sharing. It's not about keeping it to yourself. It is definitely around sharing good practice, sharing what's learnt, and also, hopefully things that went wrong for us, sharing that as well and saying this is how...don't do that, don't go down off that road because we tried that and it didn't work, as much as it is about saying this route worked really well." (Innovation directly related to the healthcare provision)

Innovators recognised that diffusion would involve adaptation to the new context. "Re-invention" [12] rather than replication was seen as an imperative for prospective adopters. In the context of complex organisational settings, interviewees stressed that to "survive" and be successful was only possible if necessary adaptations and adjustments were made.

"And also everybody, to some extent, has to evolve these things for their own circumstances, don't they?....It's not a one-size fits all." (Innovation directly related to the healthcare provision)

#### D2. Extra-organisational influences

Three main external influences were identified in innovators' accounts: (a) economic, (b) political, and (c) ideological.

##### a. Current economic climate

The constrained economic climate was often cited as inhibiting initiatives which were expensive and did not save costs directly. Such innovations were unlikely to attract funding and sustained financial support, since they were seen to oppose the measures needed in challenging economic conditions.

"I think we did it at exactly the right time, because I think, now, it's quite a difficult time in terms of obviously, the economic climate. It's really difficult in terms of funding that's available, and actually, there needed to have been some pump-priming upfront to be able to deliver this." (Innovation directly related to the healthcare provision)

##### b. Political influences

Politics was constructed both as a positive and a negative force in diffusion efforts. In terms of benefit, the presence of regulatory bodies that would shape practices around specific innovations was considered to facilitate certain initiatives. Conversely, it was more problematic where there was a more fragmented landscape of accountability and no clear responsible body to align stakeholders' activities.

"The problem is, there is actually no national body, as I said again, responsible for food. If you ring the Department of Health and say "Who's responsible for food in the NHS?" you'll get a complete blank." (Innovation less related to the healthcare provision)

Additionally, when the innovation was believed to entail political risks or ran against the dominant political forces within the local context, the diffusion met severe challenges.

##### c. Ideological influences

The last critical factor for success was the perceived fit of the innovation with the broader ideological context, both within and outside the healthcare sector. When an innovation was viewed to reflect dominant ideological beliefs and to be consistent with the "spirit of the times", initiatives were more likely to become established. This was especially so for those innovations which were peripherally linked to the core function of the NHS. In this case, innovators had to resort to ideological resources external to the domain of healthcare, such as environmentalism, in order to endorse the value of their initiatives.

"I was going to say this is a really good period of time to be doing these things because there is a general awareness, there are all sorts of things that the Trust has to do in saving energy. People think it's a good idea. The community thinks it's a good idea. It costs you if you don't." (Innovation less related to the healthcare provision)

## Discussion

This study sought to examine the subjective experiences and interpretations of factors facilitating or blocking the implementation and diffusion of process-based healthcare innovations. It did this by exploring innovators' own accounts of these processes. Overall, our results elicited themes commonly found in the literature of innovation diffusion, echoing previous studies [33,34]. Significantly, the notion of evidence consistently emerged as the key *leitmotif *in narratives of the innovation journey. The development of social networks, both inter-personal, expressed through champions and advocates, and inter-organisational, was an additional critical theme, while both the immediate organisational context and the wider socio-political and economic environment were recurrently articulated as major influencing factors.

Evidence was constructed as a powerful parameter that provided innovators with a sound base for their own assessment in turn allowing the initiation and diffusion of innovation. Evidential knowledge constituted a transparent, unbiased and credible source, from which innovators could extract their arguments and could structure their persuasive efforts in terms of innovation utility and effectiveness. As May et al. [35] suggested "evidential knowledge serves a stabilising purpose for ideational claims" (p. 703). The desire for "hard" or numerical evidence dominated. This was evident, not simply around those innovations which were more clinically-oriented but was also considered vital for innovations less centrally-related to healthcare provision or led by non-medical staff. Here too numerical or financial descriptors were aspired to; they provided ideal metrics for indicating the impact of the innovation. Importantly, a polarisation was observed with innovators often contrasting numerical evidence with experiential testimonies or anecdotal evidence, while the potential for a rigorous qualitative assessment of the value of the initiative was absent from peoples' accounts. The strong reliance on quantification and the accompanying disregard of experiential knowledge is reflective of the pursuit of objectivity. Quantitative data and the highly structured rules for producing this have been rendered as a powerful tool for conferring trustworthiness to knowledge claims, appearing exempt from subjective judgments and local singularities [36].

Although evidence was depicted as a powerful tool from the innovators' point of view, they did not claim that the evidence they had assembled were necessarily the most accurate or reliable metrics of innovation effectiveness or consider that it would be uncontested and readily acceptable among a broader range of stakeholders. Indeed, the innovators were often aware of the weaknesses or deficiencies of their approach and expressed a desire for more or stronger evidence [Vasileiou, Barnett, Young, unpublished data]. The evidence they were able to collect and produce seemed enough to convince immediate stakeholders but how compelling it was considered to be for a broader audience was much more questionable.

Arguably, the ubiquitous preoccupation with evidence reflects the strong profile of the "evidence-based practice" movement within healthcare sector since the early 1990s [37]. Though deriving from the medical community, our findings suggest that representations of evidence-based practice and of its value have been assimilated by other professional fields within healthcare. The sort of evidence perceived as adequate and thus persuasive varied considerably across the professional memberships of innovators, with medical staff espousing almost exclusively scientifically derived evidence, while other professionals contented themselves with statistics and financial figures. However, conviction concerning the necessity, transparency and objectivity of empirical data for the audit of innovations was common to all. Ultimately, evidence was constructed as the tool which would legitimate an unproblematic and direct diffusion of innovations within a sector that traditionally relied on scientific knowledge. Other sources of knowledge, such as experience, would fail to do so especially under the burden of uncertainty and risks that any organisational change embodies. Consequently, evidence constituted a stable and substantial reference point from which arguments of innovation utility could be justified and practices of persuasion could be initiated.

Several types of inter-organisational links (e.g. structural, administrative, institutional, or resource links) have been conceptualised as antecedents of organisational innovativeness [14]. In this study partnerships were not only seen as a prerequisite of innovation, but also as a result of innovation; inter-organisational links were part of the essence of the innovation itself. This was highly valued and constructed as an important "legacy" for the local community.

Our findings suggest that inter-organisational links served two important and complementary purposes: material and symbolic. Materially-based partnerships provided the innovative organisation with the necessary resources, required for the implementation and diffusion of initiatives. Symbolically, inter-organisational exchanges allowed organisations to gain local consensus and therefore to bolster the new service with legitimacy. Particularly when innovations were perceived to be radical, proactive engagement with various stakeholders was common. Consequently, inter-organisational collaboration was not only seen as vital to securing resources but also as an important social exchange that assisted with powering innovation through gaining a broader consensual base.

Reinvention [12] on the part of prospective adopters was a common theme in that innovators expected others to adapt and modify the innovation in the new context thus increasing the likelihood of sustainable diffusion. For those leading the development of new services in healthcare settings, having an identity of "successful innovator" was both feasible and desirable. It was thus vital to the maintenance of that identity that potential adopters in other healthcare organisations should understand that the initiatives were highly context-specific (see also [38]) and thus their active adoption in new contexts all but constituted another innovation.

Finally, our findings indicate the importance of a supportive environment for the establishment and diffusion of service innovation along with the technological enablers, as this is proposed in the theory of disruptive innovation [22,23]. The example of the multilateral innovation around sustainability is informative: most of the single initiatives within this innovation-green IT developments, sustainable transportation scheme-resonated with the broader spirit of environmentalism and energy-saving policies and were implemented successfully. However there was a particular case-the construction of a sustainable food procurement unit-which, even though it could bring significant cost-savings and was consonant with the sustainability agenda adopted by the Trust, was nevertheless severely impeded due to the absence of a regulatory body within healthcare that would align, support and coordinate the relevant activities.

### Limitations and strengths of the present study

One limitation of this research is its cross-sectional design which precludes an examination of the underlying processes of innovation initiation, implementation and diffusion, as a longitudinal study would have done.

However, this study provided the unique insights and experiences of healthcare innovators who had conceived and led process-oriented innovations. Innovators can contribute significantly in the diffusion of new initiatives, as they often appear willing to lead constructive efforts of dissemination, operating as powerful champions. They are able to advise and indicate the most promising ways of adoption and implementation, since they carry valuable experience of their own efforts to implement the initiative in their organisations. However attention should be paid as to what stages of diffusion innovators are more likely to contribute positively, since high levels of champions' identification with their role or organisation may actually impede further innovation diffusion [39]. On-going support of healthcare innovators, especially in their attempts to promulgate and publicise the novelty, is crucial for the dissemination of new initiatives.

## Conclusions

By interviewing key organisational representatives who had developed and established a range of healthcare service innovations from a variety of healthcare sectors we attempted to understand the factors obstructing or facilitating the innovation implementation and diffusion. A set of common determinants was identified across interviews pertaining to the availability of quantitative evidence, the building of trustworthy partnerships, the support from human resources, and the existence of a favourable inner and outer context. Innovators repeatedly stressed the necessity of innovation adaptation if it were to be implemented in a different context, suggesting innovators' awareness of the context-specific character of the innovations, and their desire to defend an identity of successful innovator. Finally, the contribution of innovators to the promulgation and dissemination of the novel message beyond the boundaries of their organisation may be beneficial in guiding and advising prospective adopters in their own effort to introduce change.

## Competing interests

The authors declare that they have no competing interests.

## Authors' contributions

JB conceived of, designed and carried out the study. JB also analysed and interpreted the data and contributed to the writing of the manuscript. KV analysed and interpreted the data and contributed to the writing of the manuscript. FD and LB carried out the study. TY conceived of and designed the study. All authors read, critically assessed and approved the final manuscript.

## Pre-publication history

The pre-publication history for this paper can be accessed here:

http://www.biomedcentral.com/1472-6963/11/342/prepub
